# Evaluation of the intensity of cigarette odors based on the perception of consumers

**DOI:** 10.18332/tpc/162103

**Published:** 2023-04-27

**Authors:** Efthimios Zervas, Niki Matsouki, Charikleia Tsipa, Emannuel Konstantinidis, Zoe Gareiou

**Affiliations:** 1 Hellenic Open University, Patra, Greece; 2 Hellenic Thoracic Society, Athens, Greece

**Keywords:** harm reduction, nicotine, prevention, odor, consumer

## Abstract

**INTRODUCTION:**

We evaluated the tobacco odor intensity of cigarettes based on a large consumer panel and explored the differences of odor intensity perception based on sex, age and smoking habits.

**METHODS:**

The perceived intensity of tobacco odor of cigarettes was evaluated using a consumer group method. A consumer panel of 240 volunteers (80 smokers, 80 ex-smokers and 80 non-smokers) was asked to smell eleven unlit cigarettes and then report their tobacco odor intensity in a specific questionnaire.

**RESULTS:**

All volunteers clearly determined the presence of tobacco odor in all cigarettes. There is a general decrease of the perceived odor intensity with age, for both males and females. Moreover, tobacco odor perceived intensity, among all volunteer groups (smokers, non-smokers, ex-smokers), was higher for females than for males. Non-smokers declared the highest perceived tobacco odor intensities, followed by ex-smokers and smokers, who recorded the lowest perceived odor intensity. Perceived odor intensity decreased with age, with a higher rate for females compared to males, but independently of the smoking habits.

**CONCLUSIONS:**

Regular and untrained consumers confirmed that a tobacco odor of different intensity can be perceived during the smelling of unlit cigarettes. This perceived intensity depends on sex, age and smoking habits.

## INTRODUCTION

Tobacco products are known for their negative health effects^1^. Flavors affect tobacco attractiveness and subsequently smoking initiation, especially among young people^2,3^. According to Article 7 of the Directive on the approximation of the laws, regulations and administrative provisions of the Member States concerning the manufacture, presentation and sale of tobacco and related products^4^, characterizing flavors in cigarettes, roll-your-own tobacco and heated tobacco products are prohibited in European Union. However, tobacco has a strong flavor itself and this flavor is one of the main parameters for the choice of a specific brand of cigarettes^5-7^. It should be noticed that the flavor of a tobacco product is a composition of several flavors, for example in the case of a cigarette, the flavor depends on the type and ratio of tobacco type used, the treatment of tobacco, the additives added, etc.

The method to assess and evaluate tobacco products’ flavors is either by a sensory experts panel or by consumer groups^8^.

The evaluation of the intensity of tobacco flavor based on expert panel can be performed using the quantitative descriptive analysis (QDA) method^9-12^. According to this method, a group of panelists is asked to quantify and identify the perceived sensory properties of a specific product. The steps followed include the development of the description language (terminology), the selection and training of the panel members, the collection of data and, finally, the analysis. During training, the panelists are asked to score the flavor intensity of the examined products using a specific scale. Quantitative flavor profile, QFP, focuses on the flavor^9^. Standards are used in order to help the panelists come to an agreement about the intensity perceived^13^, before the flavor intensity evaluation of the samples. The evaluation procedure of the samples is performed at least twice in random order^9,14^. This general procedure is also used in the methodology followed for the determination of characterizing flavors in the EU^15^.

When the evaluation is based on consumers, the panel is composed of regular consumers. The group may have specific characteristics, e.g. specific age range, if such a characteristic is required. Without any prior training, the consumers are asked to answer a questionnaire. This questionnaire initially includes questions about their sociodemographic characteristics. Next, the consumers are asked to answer several questions about their habits in relation to the product’s consumption or use. Finally, samples of the product are offered to them for evaluation and the consumers answer some questions relating to their perception of the characteristics of the specific product. The collected data are statistically analyzed at the end^16-18^. It should be noted that this method is not based on standard samples and the conclusions are based on the perception of the consumers. Assessment and evaluation of the market products’ characteristics, as perceived by regular consumers, can motivate products purchase, and thus it is considered quite useful^19,20^. Usually, 70–150 people are used in the consumers evaluation tests^17-19,21^.

Comparing the two methods, an evaluation based on a panel of experts can lead to a more rigorous and robust assessment, while that of a consumers’ panel can better reflect the real market reactions. For this reason, we used a consumers’ panel.

Beside the differences between the two different methods, human olfaction depends on several parameters, such as sex, age and smoking habits^22-26^. Some works focus on the evaluation of the flavor of tobacco products. A successfully trained panel of experts was proved, according to Krüsemann et al.^8^, a good method to assess characterizing flavors in tobacco products while Rees et al.^27^, in their review article, note the development of consumers’ sensory assessment strategies as a useful tool for tobacco industry before the production and marketing of a new product.

However, literature does not include works evaluating the tobacco flavor intensity of tobacco products using consumers and comparing the impact of sex and smoking habits (non-smokers, smokers, ex-smokers) on the perceived tobacco flavor intensity of cigarettes using commercial products.

In this work, a large consumer panel was used in order to evaluate the tobacco odor intensity of several commercial cigarettes and one research cigarette, as perceived from the members of this panel. As far as we know, evaluation of the perceived tobacco odor intensity of unlit market tobacco products has not been performed by a consumer panel. Three groups of consumers, smokers, ex-smokers and non-smokers, both males and females of all ages (adults) were asked to evaluate the tobacco odor intensity of cigarettes. No previous training was performed, so that the opinion of regular consumers is better reflected. Our primary objectives were to explore and analyze the impact of sex, age and smoking habits (non-smokers, smokers, ex-smokers) on the perceived intensity of tobacco odor of cigarettes. This objective is driven from the statement that perceived tobacco odor intensity is one of the parameters for the choice of one particular brand/type of cigarettes.

## METHODS

### Participants

The consumer panel used in this work consisted of 240 volunteers divided into three groups (80 smokers, 80 ex-smokers and 80 non-smokers), equal males and females. Smokers are active users of cigarettes (at least 10 cigarettes per day for at least 2 years and consuming only cigarettes and no other tobacco products), ex-smokers have been users in the past but have been, on the day of the test, non-smokers for at least one year, and non-smokers have never used any type of tobacco product. Ex-smokers and non-smokers were required not to be passive smokers on the day of the test. All participants were required to be in good health and have no permanent or temporary problems related to their olfaction.

The participants were recruited through multiple personal contacts and participated in this research without any kind of remuneration. Volunteers’ age varied from 19 to 66 years. All laws were followed for the individual’s protection of personal data. Since this research is not funded, no approval from the Bioethics Committee was necessary for this research according to Greek Law. Participants were free to interrupt the test at any moment and they were not exposed to any harmful tobacco emissions.

### Classification of volunteers in groups

The volunteers were classified in the following groups – ALL: all volunteers, M: males, F: females, S: all smokers, S_M: smokers males, S_F: smokers females, NS: all non-smokers, NS_M: non-smokers males, NS_F: non-smokers females, ES: all ex-smokers, ES_M: ex-smokers males and EX_F: ex-smokers females.

### Questionnaire

All volunteers were asked to answer a specific questionnaire, consisting of two parts. The first part of the questionnaire recorded the volunteer’s sociodemographic characteristics: smoking habits, sex, age, marital status, occupation status, education level, and annual income. The second part referred to the tobacco odor intensity perceived after smelling unlit cigarettes.

### Test conditions

All tests were performed from 1 April 2022 to 31 May 2022, at the premises of the Hellenic Open University. Due to restrictions in availability of the volunteers, all tests were performed in the evening (6 p.m. to 9 p.m.). Several volunteers worked simultaneously in different rooms, having all the same configuration. In order to avoid interference with probable indoor odors, the window of the test room was always open during the tests. No odors from the outdoor area were expected to interfere, since the university facilities are quite isolated from the town, with no activities around. The volunteers set on a desk and water was freely available to them. The smoker volunteers were asked not to smoke at the day of the test, and all volunteers not to be exposed to passive smoke, and avoid perfumes and spicy meals, on the day of the test.

Using a random selection between boxes, and without seeing the cigarette box and cigarette, the volunteers were asked to quantify the tobacco odor intensity of the 11 cigarettes. The cigarette was chosen by the assistant and given to the volunteer. A new box of cigarettes was used every day.

### Odor evaluation

The participants were asked to smell 11 unlit cigarettes and then report the odor intensity to the assistant, who filled in the questionnaire. Consumers were asked to evaluate only the intensity of tobacco odor, without taking into consideration any other odors, if any. The cigarette samples were from ten randomly selected brands in the Greek market (Muratti, Silk Cutsilver, Pall Mall Red, Davidoff Gold, Winston Blue, Κarelia Gold Case, R1 Red, Camel Yellow, Marlboro Red, Heets Amber) and one reference cigarette (University of Kentucky 3R4F). The odor intensity was reported on a 5-point scale: 0=no odor, 1=maybe there is an odor, 2=very low intensity of odor, 3=clear presence/medium intensity of odor, 4=strong intensity of odor, and 5=very strong intensity of odor. It should be noted that no standards were used to calibrate the scale to fixed intensities and the responses correspond only to the personal assessment of the respondents.

### Statistical analysis

After the score of odor intensity was recorded for each one of the 11 samples, the average score value and the standard deviation per volunteer was calculated. The results of the different groups were compared applying a two-side t-test and the p values were calculated. For the results of each group, a linear regression is used and the Pearson coefficient and the slope a of this regression were determined. These parameters were used in order to examine the possible correlations between age and odor perception among the different groups of volunteers. Also, a two-way ANOVA was used to determine the statistical differences between the results of the different groups.

## RESULTS

### Age characteristics of the volunteer groups

The mean age of all volunteers was 43.9 years. Table 1 shows, for each group, the mean, range, standard deviation (SD) and relative standard deviation (RSD=100×SD/mean ) of the age of each group.

**Table 1:** Main age characteristics of each group of volunteers: all volunteers (ALL), all males (M), all females (F), all smokers (S), smokers males (S_-_M), smokers females (S_-_F), all non-smokers (NS), non-smokers males (NS_-_M), non-smokers females (NS_-_F), all ex-smokers (ES), ex-smokers males (EX_-_M), and ex-smokers females (EX_-_F)

*Group*	*Mean age (range) (years)*	*SD*	*RSD*
ALL	43.90 (19–66)	11.70	26.65
M	43.83 (19–66)	11.97	27.31
F	43.97 (19–66)	11.47	26.09
S	40.86 (19–65)	11.36	27.80
S_M	41.10 (19–65)	11.37	27.67
S_F	40.62 (20–65)	11.49	28.28
NS	43.14 (19–66)	12.97	30.08
NS_M	42.85 (19–66)	13.56	31.64
NS_F	43.42 (19–65)	12.53	28.86
ES	47.70 (27–66)	9.60	20.13
ES_M	47.55 (27–66)	10.07	21.18
ES_F	47.85 (29–66)	9.24	19.31

RSD: relative standard deviation.

The mean and range of the age of males and females, and also the SD and RSD values, are quite close for all volunteer groups. Mean and minimum age of the groups based on smoking habits is higher for the group of ex-smokers, as expected. Ex-smokers have a mean age of 47.70 years compared to 40.86 years and 43.14 years for smokers and non-smokers, respectively.

### Impact of sex and smoking habits on the perceived odor intensity

All volunteers reported an intensity value higher than 0 for all products. The mean value of the odor intensity of all products perceived by the volunteers was 3.01 in the 0–5 scale used here, with a standard deviation of 0.57, or 18.83%.

**Figure 1:**
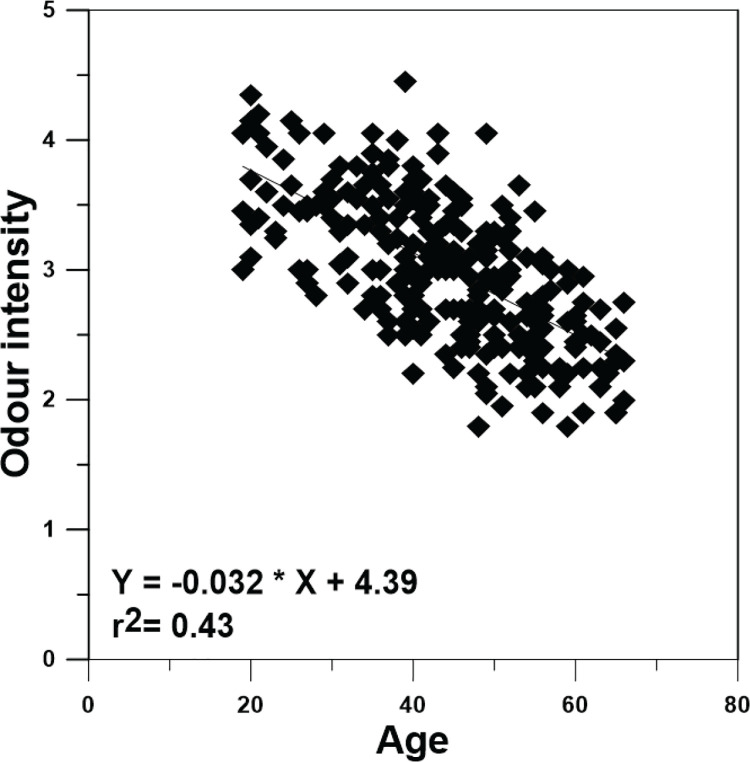
Odor intensity mean value of all categories

Figure 1 shows the mean value of the perceived odor intensity for all volunteers and also in relation to their sex, to their smoking habits and to both their sex and smoking habits. It can be observed that the females’ score is slightly higher than the score of males (3.12 vs 2.89), as already reported in the literature^24,26,28^. In our case, females report, on average, 1.08 times higher odor perceived intensity values than males (1.08=ratio of scores of 3.12/2.89). Literature suggests that the difference between males and females for the reported perceived odor intensity is not significant^29^; however, a two-side t-test showed in our study that there is a statistical difference between the scores of the two sexes (p<0.05). The values of the standard deviation of the perceived intensity of males and females are found to be quite close: 0.51 (17.75% of the mean value) and 0.59 (19.07% of the mean value), respectively.

As far as the impact of smoking habits on odor intensity perception is concerned, Figure 1 shows that the perception of odor intensity has the highest value in the case of non-smokers (3.19), followed by ex-smokers (3.00); while smokers record the lowest value (2.83). This ranking is in accordance with previous findings^22,30^. Α two-side t-test showed that there is a statistical difference between the scores of the three groups, taken by pairs (S/NM, S/ES, NS/ES) (p<0.05). The values of the standard deviation of the perceived odor intensity of smokers, non-smokers and ex-smokers are quite close: 0.55 (19.34% of the mean value), 0.61 (19.03%) and 0.49 (16.30%), respectively.

In order to investigate whether the perceived odor intensity is affected by sex and smoking habits, a supplementary statistical test, a two-way ANOVA, was used.

For the application of the two-way ANOVA, the perceived odor intensity was considered as a dependent variable and smoking habits and sex as independent variables. The level of statistical significance was set at p<0.05. The result of the two-way ANOVA, i.e. whether any of the two independent variables or their interaction is statistically significant is shown in Table 2.

**Table 2:** Correlations between perceived taste intensity and sex (males, females) and smoking habits [smokers (S), non-smokers (NS) and ex-smokers (EX)], applying two-way ANOVA

*Source*	*Type III sum of squares*	*df*	*Mean square*	*F*	p*
Corrected model	8.139	5	1.628	5.558	0
Intercept	2170.814	1	2170.814	7411.568	0
Sex	3.128	1	3.128	10.68	0.001
S_NS_EX	5.007	2	2.504	8.548	0
Sex × S_NS_EX	0.004	2	0.002	0.006	0.994
Error	68.538	234	0.293		
Total	2247.49	240			
Corrected total	76.676	239			

* The level of statistical significance was set at p<0.05.

The results show that the independent variables (sex and smoking habits) have a statistically significant impact on the dependent variable (perceived odor intensity) (F=10.680, p=0.001 and F=8.548, p=0.000, respectively). That is, there is a difference in the perceived odor intensity: 1) between all males and all females, and 2) between all smokers, all non-smokers and all ex-smokers.

Figure 1 shows that the average values of perceived tobacco odor, based on both smoking habits and sex, present the same trends as already described. Smokers males record a lower value than smokers females, ex-smokers males lower than ex-smokers females, and the same is found for non-smokers males and females. However, the two-way ANOVA shows that for the interaction of sex and smoking habits there is no statistically significant interaction (F=0.006, p=0.994). That is, the perceived odor intensity for the three categories (smokers, non-smokers and ex-smokers) is not statistically different for males and females. Smokers, non-smokers and ex-smokers present the same perceived odor intensity regardless of whether they are males of females.

### Impact of age on the perceived odor intensity

**Figure 2:**
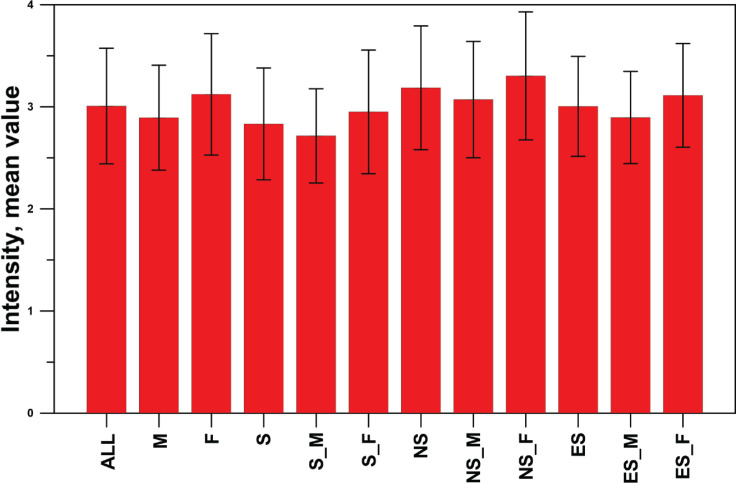
Odor intensity evaluation with age for all volunteers

Figure 2 presents the mean value of the odor intensity for all volunteers, in relation to their age. This figure shows that the score of odor intensity decreases with age, which is in agreement with the observed decrease of olfaction function with age^24,26,31^.

**Figure 3:**
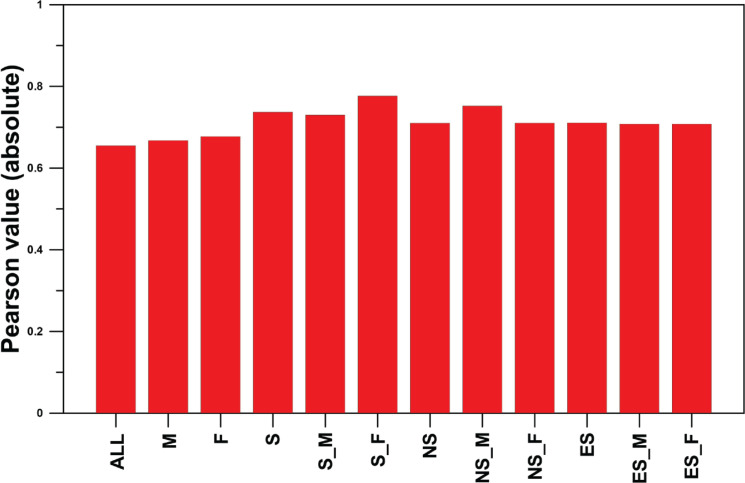
Pearson coefficient absolute values of all categories

**Figure 4:**
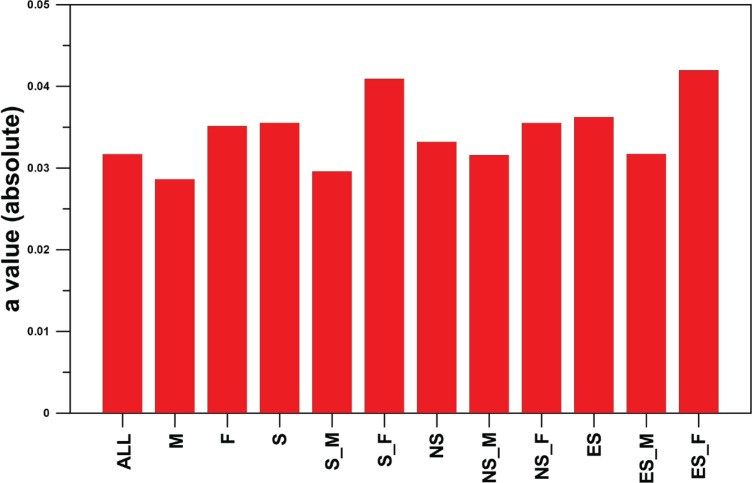
Coefficient a absolute values of all categories

The evolution of the perception of odor intensity with age was statistically analyzed. To examine the correlation of odor intensity and age, the Pearson coefficient was used. To better represent these values, Figures 3 and 4 show the absolute values of these parameters.

Figure 3 presents the absolute values of the Pearson coefficient for all volunteers in relation to sex, to their smoking habits and to both their sex and smoking habits. A Pearson absolute value >0.7 indicates a strong correlation between the two variables. Pearson values are negative, confirming that odor intensity decreases with age for both sexes and independently of the smoking habits of the volunteers.

Figure 4 shows the absolute values of the slope *α* of the linear regression of odor intensity with age. The value of *α* is -0.029 for males and -0.035 for females, -0.036 for both smokers and ex-smokers and, slightly lower, -0.033, for non-smokers, indicating that the perception of odor intensity decreases for both sexes and independently of the smoking habits with age, but the rate of this decrease is slightly higher in the case of females and in the case of smokers and ex-smokers compared to non-smokers.

### Impact of other parameters on the perceived odor intensity

The correlation coefficients between the perceived intensity of tobacco odors and the other personal data recorded including marital status, occupation status, education level and annual income, were computed. The results show that the correlation is poor, indicating that these parameters have no impact on the perceived intensity of tobacco odors.

## DISCUSSION

In this work, the evaluation of tobacco odor intensity was performed in the absence of standards. No standards or sniffing sticks were used at any point. The perceived tobacco odor intensity of randomly selected panelists was studied and analyzed.

According to regular and untrained consumer’s assessment, it is confirmed that a tobacco odor of different intensity can be perceived during the smelling of unlit cigarettes.

The human sense of smell, olfaction, which is responsible for the perception of odors in the environment, such as the odor of food and drinks, flowers and perfumes, personal care products, nature etc., depends on several parameters and is subject to alterations during a human’s life. A number of studies have shown a decrease in the olfaction function with age^24,26,28^. The rate of smell deterioration with age is logarithmic and corresponds to a loss of 50% of smell sensitivity in 22 years^28^. The Pennsylvania smell identification test suggests alterations of the olfaction initiating after the age of 60 for males and 70 for females^31^. The present study suggests that not only the olfaction decreases with age, but also the perceived intensity decreases, and this can be a result of the decrease in olfactory acuity. Furthermore, it was found that the rate of decrease of the perceived tobacco odor intensity is not the same between males and females. Though females scored higher on odor intensity perception, decrease happens with a higher rate for them in comparison to males.

In relation to sex, females are considered to have superior olfaction function compared to males, but the differences reported in the literature between the two sexes are not significant^29,32^. Our work confirms that there is an impact of sex on the perceived odor intensity, with females of all ages and groups scoring slightly, but consistently, higher than males. In disagreement with previous findings, our results show a statistically significant difference between the two sexes.

Concerning the effect of smoking on olfaction, literature suggests that smoking has a negative effect on olfactory sensitivity, making smokers less capable to recognize and discriminate flavors and also making them have a lower intensity perception^22^. Furthermore, the negative impact of smoking on olfactory functions is comparable for both active and passive smokers^33^. Chronicity and level of dependence on smoking is proposed to be associated with the rates of alterations in the sense of smell^23^. However, even in that case, smoking cessation can improve odor perception^30^, and ex-smokers do not present increased risk of olfaction dysfunction compared to non-smokers^34^. It is still unclear how soon olfactory dysfunction can improve after quitting smoking. Da Ré et al.^30^ suggest that this happens very quickly while Siegel et al.^35^ reported that the decreased ability of odor recognition, evaluated using sniffing sticks, olfactory persisted for at least 15 years after quitting. Still, there are a few studies that suggest no correlation between smoking and odor recognition^32^. Our work confirms that there is a degradation of the perceived olfactory function due to smoking. The extent to which olfaction is affected should be further analyzed based on the years of smoking, and the number of cigarettes smoked per day. Since ex-smokers scored better than smokers but worse than non-smokers, it can be suggested that the negative impact of smoking on odor perception ability is partially reversible. Further studies should be performed focusing on ex-smokers odor intensity perception in relation to the number of years after quitting smoking in order to ascertain if olfaction degradation can be completely reversed after longterm smoking abstinence.

The fact that the rate of decrease in odor perception intensity with age was the same for smokers and ex-smokers and slightly better for non-smokers implies that the odor perception is not significantly affected by smoking habits and that the decrease will inevitably take place with age, independently of the smoking habits, but strongly depended on sex.

### Limitations

Some limitations of this work should be mentioned. Firstly, only 11 market products were included. Though randomly selected, the results may not be absolutely representative of all the products available in the Greek market. The study should be extended to include more brands. Secondly, there is no evaluation of reference samples, so the results are based on the consumers perception. Reference samples should be included in future research in order to enable initial intensity scale calibration, using a trained panel.

Further work should also be extended to include different kinds of tobacco products, such as electronic cigarettes and heated tobacco products. Moreover, odors discrimination and evaluation of the intensity of the different odors could be performed as well, additionally to the tobacco odor intensity evaluation. Also, a more global application of this research should be performed, as different ethnic groups may have a different response to the perceived odor intensity.

## CONCLUSIONS

This work evaluated the perceived tobacco odor intensity of a number of commercial and one reference cigarette, based on a group of volunteers. The main results show that there is a general decrease of the perceived odor intensity with age, for both males and females. Sex also affects odor perception, among all volunteer groups (smokers, non-smokers, ex-smokers), as females score slightly higher than males. Non-smokers report, on average, the highest tobacco odor perceived intensities, followed by ex-smokers and smokers, who record the lowest tobacco odor perceived intensity. The lowest tobacco odor perception of smokers confirms that smoking has a negative impact on olfaction.

## Data Availability

The data supporting this research are available from the authors on reasonable request.
